# Associations of homelessness and residential mobility with length of stay after acute psychiatric admission

**DOI:** 10.1186/1471-244X-12-121

**Published:** 2012-08-21

**Authors:** Alex D Tulloch, Mizanur R Khondoker, Paul Fearon, Anthony S David

**Affiliations:** 1 King’s College London, King’s Health Partners, Institute of Psychiatry, De Crespigny Park, London, SE5 8AF, UK; 2 NIHR Specialist Biomedical Centre for Mental Health, Maudsley Hospital, London, SE5 8AZ, UK; 3 Trinity College Dublin and St Patrick’s University Hospital Dublin, James’s Street, Dublin 8, Eire

**Keywords:** Length of stay, Hospitals psychiatric, Mental disorders, Residential mobility, Homeless persons

## Abstract

**Background:**

A small number of patient-level variables have replicated associations with the length of stay (LOS) of psychiatric inpatients. Although need for housing has often been identified as a cause of delayed discharge, there has been little research into the associations between LOS and homelessness and residential mobility (moving to a new home), or the magnitude of these associations compared to other exposures.

**Methods:**

Cross-sectional study of 4885 acute psychiatric admissions to a mental health NHS Trust serving four South London boroughs. Data were taken from a comprehensive repository of anonymised electronic patient records. Analysis was performed using log-linear regression.

**Results:**

Residential mobility was associated with a 99% increase in LOS and homelessness with a 45% increase. Schizophrenia, other psychosis, the longest recent admission, residential mobility, and some items on the Health of the Nation Outcome Scales (HoNOS), especially ADL impairment, were also associated with increased LOS. Informal admission, drug and alcohol or other non-psychotic diagnosis and a high HoNOS self-harm score reduced LOS. Including residential mobility in the regression model produced the same increase in the variance explained as including diagnosis; only legal status was a stronger predictor.

**Conclusions:**

Homelessness and, especially, residential mobility account for a significant part of variation in LOS despite affecting a minority of psychiatric inpatients; for these people, the effect on LOS is marked. Appropriate policy responses may include attempts to avert the loss of housing in association with admission, efforts to increase housing supply and the speed at which it is made available, and reforms of payment systems to encourage this.

## Background

The length of stay (LOS) of psychiatric inpatients continues to be highly variable, despite a trend towards overall reduction in most developed countries [1]. For example, an analysis of English psychiatric hospital admissions in 1999–2000 demonstrated that median LOS was 15 days, but 9% of admissions lasted 90 days or more, and 1% lasted a year or more [2]. Numerous studies have attempted to explain this variation, which arises both at individual patient level and also at provider level [3]. Our systematic review of the many studies on LOS in US mental health services [4] indicated that psychosis and female gender are associated with increased LOS, while discharge against medical advice, prospective payment, being married, being detained and either younger or middle age are associated with decreased LOS. However, the proportion of variance in LOS explained in these studies was rarely greater than 20-30%. Although the mechanisms by which these factors influence LOS tend not to be discussed in detail, our presumption is that any associations need to be explained with reference to the behaviour of health care professionals.

A group of factors that have been little explored in studies of LOS are variables related to housing and homelessness. The importance of such factors is two-fold: they may serve to explain additional variation in LOS, and, more importantly, they are factors which at least in principle could be addressed by practical measures to increase the availability of housing and the speed with which it is made available. During the preparation of our systematic review [4], we found only two studies which examined such factors among the 106 that we screened in full for inclusion (see below); similarly, housing-related factors were neither examined in previous high-quality studies of LOS in England [5,6], Germany [7] and Scandinavia [8], nor in case–control studies of long-stay versus typical length admissions performed in the US [9] and in Switzerland [10,11]. Studies of LOS which have looked at housing-related factors include a large Swiss analysis [12], which found that living conditions (including homelessness) was one of several variables strongly associated with LOS; an Australian study of LOS based on measures including the Health of the Nation Outcome Scales (HoNOS; [13]), which found a strong positive association with the HoNOS problems with living conditions item [14]; a large study of LOS in New York [15], which found a strong positive association with homelessness; a Californian study of a Veterans’ Administration Health Center with access for a “hoptel” for homeless patients enabling early discharge, which found, in contrast to the New York findings, and presumably due to the study intervention, that LOS for homeless patients did not differ from those who were housed [16]; and our own small case–control study of “long-stay” patients in psychiatric services serving four South London boroughs which found that long-stay was associated with need for rehousing [17].

The relative infrequency with which these variables have been examined in studies of LOS contrasts with their prominence in the UK literature on “delayed discharge” [18,19]. Hospital admissions categorised as delayed discharges (or delayed transfers of care) are those which are judged to have been prolonged beyond that which would have been necessary in the absence of the factor responsible for the delay. Studies of delayed discharge have been performed both in samples of long-stay patients and in prevalent samples of psychiatric inpatients. A study of 15 mental hospitals in England and Wales during 1972 and 1973 found that one third of those in hospital for between 1 and 3 years could have been discharged had suitable accommodation been available [20]. A similar UK-wide study performed in 1992, after 20 years in which the number of psychiatric beds nationally had halved, found that 61% of those who had been in hospital for between 6 months and 1 year were considered by the treating clinician to be more appropriately cared for outside hospital [21]. A further study of two small cohorts of long-stay patients found that 60% did not need to be in hospital and would have been discharged had suitable accommodation been available [22]. Among various prevalent samples of psychiatric inpatients, the proportion classified as being a delayed discharge either due to lack of housing or to which lack of suitable housing contributed was 356/227 (15%) [23], 396/3710 (11%) [24] in two London-based studies; and 289/2236 (13%) [25] in a national survey.

An advantage of attempting to examine variation in LOS through the prism of delayed discharge is that this concept focuses on the effect of the cause of the delay on clinical decision-making and the practical consequences of ameliorating the cause of the delay, and therefore explicitly relates longer hospital stay to decisions made by staff in relation to some defined problem, be that housing-related or due to delay in arranging a suitable package of care, etc. However, defining a delayed discharge depends on a subjective judgment of what would have happened to a patient in the absence of the putative delaying factor, and, furthermore, this method cannot be symmetrically applied to other factors which may influence LOS—it is likely to be meaningless or impractical to imagine, for example, what a patient’s LOS might have been had they had a different mental illness or other individual characteristic. Therefore, we consider that it is preferable to study LOS itself, and to examine the association between LOS and the factors thought to be responsible for delayed discharge alongside other factors. In the case of delay due to housing-related issues, we suggest that healthcare professionals tend to delay the discharge of patients who are homeless—because such patients either need rehousing or at least need to be better stabilised before returning to tenuously held accommodation—and that they also tend to delay the discharge of patients who are anticipated to move residence (if the new residence is assumed to be more suitable on health grounds). It follows that we should seek to estimate the associations between LOS and *homelessness* and between LOS and *residential mobility*—the latter is defined here as moving from one address to another or, if previously homeless, moving into a new address, and is understood as being associated with LOS because of the anticipatory effects referred to above.

Homelessness is well-described among psychiatric inpatients [26-35], while hospital admission is the most consistently replicated association with residential mobility among those with mental illness [36-42]. That homelessness and residential mobility have been so little examined in the literature on LOS probably reflects their being infrequently included in sets of routinely-collected data. The present study is based on a dataset of discharges after acute psychiatric admission from hospitals operated by South London and Maudsley NHS Trust and serving the London Boroughs of Croydon, Lambeth, Lewisham and Southwark. We have previously published cross-sectional analyses of the associations of homelessness and of residential mobility in this sample [43,44], finding that 16% of admissions were associated with homelessness, which tended to be present at admission or to be first recorded shortly afterwards, and that 15% of admissions were associated with residential mobility during the admission or up to 28 days after discharge, with residential mobility tending to be recorded around the time of discharge. Although homelessness and residential mobility were strongly associated, the association was not invariable, with around half of homeless individuals not recorded as moving into new accommodation. We aimed to estimate the associations between LOS and homelessness, residential mobility and other factors, and to estimate the extent to which variation in LOS was accounted for by each of these factors.

## Methods

Data came from the BRC Case Register, which is an anonymised copy of the South London and Maudsley NHS Foundation Trust's paperless electronic patient record database, optimised for data extraction [45] and maintained by the Trust’s Biomedical Research Centre (BRC). The Case Register covers all mental health services for much of South London since 2006 and includes structured clinical and administrative data (the main source of data for this analysis) and free-text data. Access to the Case Register is restricted to researchers approved by the BRC, and the BRC’s Oversight Committee also granted permission for the present study. Ethical approval is not required as data are fully anonymised.

Details of the sample were previously described [43,44]. We initially extracted all psychiatric hospital stays leading to a discharge between 31st December 2007 and 31st December 2009. From these, we selected all hospital stays that had begun on an acute psychiatric ward. (The sample therefore included a small number of individuals who were admitted to acute psychiatric wards, then later transferred to rehabilitation or forensic services, and then discharged.) When the same individual had more than one discharge over this period, the last hospital stay was selected; this avoided clustering effects incompatible with the multiple imputation model used. Length of stay was defined as the difference between the discharge date and admission date in days.

Additional data merged with these were: age, sex, ethnicity, marital status, employment status, recorded ICD-10 diagnoses, most restrictive legal status in the seven days after admission, number of psychiatric ward discharges in the two years preceding the index admission date, longest psychiatric admission leading to a discharge in the two years preceding the index admission date, whether any hospital admission leading to discharge in the two years preceding the index admission date had included a period of psychiatric intensive care, admission HoNOS scores, and data describing periods of homelessness or resident in a particular “output area” (these comprise around 500 households). Legal status was classified as informal, Section 2 (which permits detention for up to 28 days), or Section 3 / Forensic (detention for up to six months in the first instance). The nearest multi-axial diagnosis to the date of discharge was selected. A preliminary analysis of the LOS of non-comorbid cases was used to define a diagnostic hierarchy; this was then used to define primary diagnosis if necessary. Variables were also created for lifetime drug and alcohol diagnosis and for residential mobility. Imputing large amounts of data using multinomial logistic regression is computationally intensive and very slow: therefore HoNOS item scores (except problems with living conditions) were recoded as “low” (0–1) or “high” (2–4).

We used Stata version 12 for analysis. Initially, we performed descriptive and unadjusted analyses. After exploring missing data, we then used multiple imputation using chained equations [46,47] to impute the missing values. In addition to the variables listed above, the imputation model included additional predictors of missingness (see Results), and the Nelson-Aalen cumulative hazard estimator for discharge, taken from a Cox regression of LOS fitted without covariates. The number of imputed datasets created (50) was decided on using the rule that this should equal or exceed the percentage of missing values for the variable with the greatest number of these [48], and imputed values were compared with original values.

After transforming LOS to its logarithm, we used linear regression to model the statistical effects of exposure and to estimate both the proportion of variance explained overall (R^2^) and incremental effects on R^2^ (the difference in R^2^ for the final model and a model without the test variable). Although LOS data may be more flexibly modelled using survival analysis, linear regression has the advantage of being consistent with most previous research and of readily yielding estimates of R^2^.

An appropriate functional form for continuous variables (age and longest admission in the preceding two years) was defined using the method of fractional polynomials [49,50], using a dataset with complete data for most important exposure variables (demographics, diagnosis, legal status and housing variables). Subsequent model-building was performed using the imputed datasets, using the Stata mim procedure to combine parameter estimates as per “Rubin’s rules” [51]. All exposure variables listed above were included in the full model, with the exception of the HoNOS problems with living conditions item, which was highly correlated with residential mobility and homelessness, and was therefore omitted. Following the empirical approach outlined by Hosmer et al. (2008) [52], we first fitted the full model as above, then removed all variables for which the incremental F test gave p > 0.25, and then removed other variables with p > 0.05 one-by-one, seeking to retain only those variables whose removal led to substantial change in other coefficients (approximately 20% or more) and which, when added to the final model, remained insignificant at the p = 0.05 level. Estimates of average R^2^ were generated as described by Harel (2009) [53]. An estimate of overall R^2^ for the final model was obtained. In order to explore the final model further, estimates of incremental R^2^ were calculated by subtracting the estimated R^2^ for the regression without one or more variable(s) from the overall R^2^ for the final model.

## Results

There were 4485 admissions. Mode and median LOS were 1 day and 22 days respectively; LOS exhibited a strong right skew (4.6; p < 0.0001). This skew was largely abolished by log transformation (0.09; p =0.0186), and asymmetry was reduced. Sample characteristics are tabulated in Table 1. Ten subjects were under 18 and 114 (3%) were over 65. Values of age were missing for 3 subjects, ethnicity for 92 (2%), address data for 99 (2%), marital status for 355 (8%) and diagnosis for 393 (9%). There were more missing data for employment (42%) and for HoNOS item scores which, for example, were 25% missing in the case of HoNOS ADL impairment. Missingness for HoNOS items was related to the date of admission, eventual LOS, and the first ward to which the subject was admitted. Other data were complete.

**Table 1:** Sample characteristics

**Variable**		**Complete (% / total)**	**Measure**	
Age		4482 (99.9%)	Mean (SD)	39.3 (12.3)
Gender	Male	4485 (100%)	N (%)	2514 (56.1%)
	Female			1971 (44.0%)
Ethnicity	White British	4393 (98.0%)	N (%)	1710 (38.9%)
	Black African or Caribbean			1730 (39.4%)
	Other			953 (21.7%)
Marital status	Single	4130 (92.1%)	N (%)	2999 (72.6%)
	Divorced / separated / widowed			603 (14.6%)
	Married			528 (12.8%)
Employed	No	2604 (58.1%)	N (%)	2316 (88.9%)
	Yes			288 (11.1%)
Diagnosis	Drug & alcohol	4092 (91.2%)	N (%)	370 (9.0%)
	Non-psychotic			1175 (28.7%)
	Other psychotic ^a^			1285 (31.4%)
	Schizophrenia			1262 (30.8%)
Legal status ^b^	Informal	4485 (100%)	N (%)	2491 (55.5%)
	Section 2 MHA			1178 (26.3%)
	Section 3 & Forensic MHA			816 (18.2%)
Longest admission in preceding two years	Nil	4485 (100%)	N (%)	2630 (58.6%)
	1-16 days			477 (10.6%)
	17-39 days			455 (10.1%)
	40-89 days			463 (10.3%)
	90+ days			460 (10.3%)
Number of discharges in preceding two years	Nil	4485 (100%)	N (%)	2630 (58.6%)
	1			638 (14.2%)
	2			522 (11.6%)
	3			253 (5.6%)
	4			170 (3.8%)
	5 or more			272 (6.1%)
HoNOS #1 (Overactive, aggressive, disruptive or agitated behaviour)	Nil or minor (0-1)	3429 (76.5%)	N (%)	2015 (58.8%)
	Mild to very severe (2-4)			1414 (41.2%)
HoNOS #2 (Non-accidental self-injury)	Nil or minor (0-1)	3420 (76.3%)	N (%)	2684 (78.5%)
	Mild to very severe (2-4)			736 (21.5%)
HoNOS #3 (Problem drinking or drug-taking)	Nil or minor (0-1)	3353 (74.8%)	N (%)	2351 (70.1%)
	Mild to very severe (2-4)			1002 (29.9%)
HoNOS #4 (Cognitive problems)	Nil or minor (0-1)	3401 (75.8%)	N (%)	2621 (77.1%)
	Mild to very severe (2-4)			780 (22.9%)
HoNOS #5 (Physical illness or disability problems)	Nil or minor (0-1)	3400 (75.8%)	N (%)	2767 (81.4%)
	Mild to very severe (2-4)			633 (18.6%)
HoNOS #6 (Problems associated with hallucinations and delusions)	Nil or minor (0-1)	3397 (75.8%)	N (%)	1439 (42.4%)
	Mild to very severe (2-4)			1958 (57.6%)
HoNOS #7 (Problems with depressed mood)	Nil or minor (0-1)	3407 (76.0%)	N (%)	1949 (57.2%)
	Mild to very severe (2-4)			1458 (42.8%)
HoNOS #8 (Other mental and behavioural problems)	Nil or minor (0-1)	3425 (76.4%)	N (%)	1475 (43.1%)
	Mild to very severe (2-4)			1950 (56.9%)
HoNOS #9 (Problems with relationships)	Nil or minor (0-1)	3332 (74.3%)	N (%)	1814 (54.4%)
	Mild to very severe (2-4)			1518 (45.6%)
HoNOS #10 (Problems with activities of daily living)	Nil or minor (0-1)	3364 (75.0%)	N (%)	2179 (64.8%)
	Mild to very severe (2-4)			1185 (35.2%)
HoNOS #11 (Problems with living conditions)	Nil (0)	3187 (71.0%)	N (%)	1609 (50.5%)
	Mild (1)			621 (19.5%)
	Moderate (2)			434 (13.6%)
	Moderately severe (3)			247 (7.8%)
	Severe to very severe (4)			276 (8.7%)
HoNOS #12 (Problems with occupation and activities)	Nil or minor (0-1)	3217 (71.8%)	N (%)	1938 (60.2%)
	Mild to very severe (2-4)			1279 (39.8%)
Homeless	Not homeless	4386 (97.8%)	N (%)	3667 (83.6%)
	At admission			392 (8.9%)
	Day 1 onwards			327 (7.5%)
Residential mobility during admission or within 28 days of discharge	No	4386 (97.8%)	N (%)	3740 (85.2%)
	Yes			646 (14.7%)

*Note.* Total sample size was 4485.

^a^‘Other psychotic’ comprised ICD-10 codes F21 to F31 inclusive. ^b^Legal status was defined as the most restrictive section of the Mental Health Act in force during the first week of the admission. Detention only under Section 136, Section 5(2) or Section 5(4) was treated as informal legal status.

No anomalies were found in the imputed data. Appropriate fractional polynomial transformations were used for age (which was entered as age cubed) and longest admission in the preceding two years (which was entered as its logarithm, together with a dummy variable representing having had no discharge from a psychiatric ward in the two years preceding the index admission date—this was necessary in order to properly fit the regression model to data including a large proportion of zero values [50]).

Seven subjects with outlying values for LOS were omitted from the regression analysis. After fitting of the initial multivariable model, the incremental F test gave p > 0.25 for the following variables, which were therefore removed: sex, ethnicity, employment, number of discharges from a psychiatric ward in the two years preceding the index admission date, lifetime diagnosis of a drug and alcohol disorder, the HoNOS overactive, aggressive, disruptive or agitated behaviour item, the HoNOS other mental or behavioural problems item, the HoNOS problems with relationships item and the HoNOS problems with occupation and activities item. Marital status and the HoNOS problem with drinking or drug-taking item were initially retained, but were removed during the subsequent model building stages (p = 0.06 for addition of marital status to the final model; p = 0.12 for addition of the HoNOS problems with drinking or drug-taking item).

In the final model, LOS was increased by residential mobility (p < 0.001), homelessness (p < 0.001), diagnosis other than drug and alcohol disorder (in ascending order of magnitude: non-psychotic disorders, other psychotic disorder, then schizophrenia; p < 0.001 for each); detained legal status (in ascending order of magnitude: Section 2, then Section 3 or forensic sections; p < 0.001 for both); and a high score on the following HoNOS items: cognitive impairment (p = 0.015), physical illness or disability (p = 0.005), hallucinations and delusions (p = 0.023), depressed mood (p = 0.028) and ADL impairment (p < 0.001). LOS was reduced by a high score on the HoNOS self-harm item (p < 0.001). As noted above, there were non-linear effects of age (p < 0.001) and length of the longest admission in the preceding two years (p < 0.001). Having a short admission in the preceding two years was associated with the shortest LOS; having a long admission with the longest; the effect of no admission was intermediate. LOS was longest for older patients, but the effect of age reduced with decreasing age. In the results table (Table 2), category-based estimates were generated for these continuous covariates, so that the effects presented are the average effects estimated for bands of values [54]. Results are expressed in their exponentiated form (exp β); therefore they represent multiplicative effects on untransformed values of LOS.

**Table 2:** Linear regression model

**Variable**		**exp β (95% CI)**	**incremental R**^**2**^	**P**
Residential mobility		1.99 (1.80,2.20)	0.028	<0.001
Homelessness		1.45 (1.32,1.60)	0.009	<0.001
Legal status^1^			0.082	<0.001
	Informal	1		
	Section 2	1.86 (1.71,2.02)		
	Section 3 or Forensic	3.06 (2.78,3.38)		
Age / years ^2^			0.003	<0.001
	16-25	1		
	26-35	1.00 (1.00,1.00)		
	36-45	1.02 (1.01,1.03)		
	46-55	1.08 (1.04,1.12)		
	56-65	1.20 (1.10,1.30)		
Diagnosis			0.029	<0.001
	Drug and alcohol	1		
	Non-psychotic	1.35 (1.18,1.53)		
	Other psychotic	1.88 (1.65,2.15)		
	Schizophrenia	2.33 (2.03,2.67)		
Longest admission in preceding 2 years ^3^	None	1	0.011	<0.001
	1-16 days	0.98 (0.89,1.07)		
	17-39 days	1.18 (1.10,1.26)		
	40-71 days	1.31 (1.22,1.41)		
	90+ days	1.53 (1.39,1.68)		
HoNOS #2	Mild to very severe	0.79 (0.72,0.87)	0.004	<0.001
HoNOS #4	Mild to very severe	1.14 (1.03,1.27)	0.002	0.015
HoNOS #5	Mild to very severe	1.16 (1.05,1.29)	0.002	0.005
HoNOS #6	Mild to very severe	1.10 (1.01,1.20)	0.001	0.023
HoNOS #7	Mild to very severe	1.11 (1.01,1.21)	0.001	0.028
HoNOS #10	Mild to very severe	1.38 (1.27,1.49)	0.011	<0.001

*Note.* N = 4478 after subtraction of seven observations with outlying values for LOS. Overall r2 was 0.368. ^1^ Legal status was defined as the most restrictive section of the Mental Health Act in force during the first week of the admission. Those detained only under Section 136, Section 5(2) or Section 5(4) were treated as informal. ^2^ Exponentiated coefficients for age-band are category-based estimates derived from the full regression model. Reference points for age used to generate these estimates were 20, 30, 40, 50 and 60 years. ^3^ Reference points for the category-based estimates for the effect of length of the longest previous admission were the median values of log previous LOS in each band.

The overall value of R^2^ for the final model was 0.37. The incremental R^2^ was greatest for legal status (0.08). For residential mobility, incremental R^2^ was 0.03 and for homelessness it was 0.01. In comparison, the value for diagnosis was 0.03; for longest previous admission it was 0.01; for the HoNOS activities of daily living item it was 0.01; and both for all the other included HoNOS items together and for age it was less than 0.01.

## Discussion

We performed a large study of associations with LOS after acute psychiatric admission, aiming to estimate the strength of association between LOS and residential mobility, homelessness and other variables. Both residential mobility and homelessness were strongly associated with increased LOS: the regression analysis indicated that LOS was on average 99% higher for residential mobility and 45% higher for homelessness. The only parameter estimates that were greater were for the effect of schizophrenia compared to primary drug and alcohol disorder and for the effect of detention under Section 3 or a forensic section compared to informal legal status. The overall value of R^2^ (0.37) was higher than many other studies of large samples of admissions, perhaps because we were able to study several variables in addition to those which have usually been studied; the greatest contribution was made by legal status, but the contribution of residential mobility and homelessness together was greater than that of diagnosis. The contribution of other variables was very modest, although the contribution of the HoNOS item scores (and especially that of the HoNOS activities of daily living item) are likely to have been reduced by the need to recode them as dichotomous variables. The contributions of homelessness and residential mobility are particularly notable given that these exposures were present in only 16% and 15% of cases respectively.

### Comparison with previous studies

Most large US studies have found effects of diagnosis and age; some have also described interactions between age and diagnosis, which we did not test for [55,56], and non-linear effects of age [57,58]. US studies have typically found a small increase in LOS for female patients which we did not observe [57-63]. The large effects of legal status are opposite to those in the US, where civil commitment procedures are typically of shorter duration, and a spike in discharge may be demonstrated at the end of the committed period [62], but they are similar to those observed in UK data collected in the 1980s and early 1990s [5].

Effects of previous service use have been inconsistent in the US literature [4]. We demonstrated a highly significant relationship between LOS and the length of the longest admission in the preceding two years. In contrast, there was no effect of the absolute number of previous discharges, although modelling the effect of previous LOS did require the additional inclusion of a dummy variable for having had no hospital admission in the relevant period. The novel finding that patients who had not had a previous admission had LOS intermediate between those with a short previous stay and a long previous stay is presumably explained by an underlying dimension of illness severity which influences a person’s past, current and future LOS. In the group with no previous admission, there is no outward expression of this underlying dimension; furthermore, individuals within this group would also be expected to exhibit the entire range of severity, with the overall effect observed therefore representing an averaging process.

We found no effect of drug and alcohol use comorbid with primary mental disorder (as measured by HoNOS and lifetime ICD-10 diagnosis), although a primary diagnosis of drug and alcohol use was strongly associated with shorter LOS. Findings in relation to this effect of “dual-diagnosis” in smaller US studies of unselected psychiatric patients have been conflicting [4], but a similar lack of consistent effect was reported in a study of over 20,000 schizophrenia and affective psychosis discharges in Arizona, California and Maryland [64]. In relation to the HoNOS items, the unadjusted effect of the problems with living conditions item and the adjusted effect of the self-harm, depressed mood and ADL impairment and items are consistent with the Australian study cited above [14], but we did not replicate the associations found with the aggressive behaviour item. Differences between this and our study may reflect differences in the construction of the regressions, different coding of the diagnosis item, the inclusion of legal status in our analysis, and greater statistical power of our analysis. We suggest that these effects may readily be interpreted in terms of their effect on the quantity of care required; this includes the effect seen of suicidality, where the reduced LOS presumably reflects the typically rapid resolution of such symptoms relative to other symptoms, with consequent discharge.

### Limitations

Our data derive only from a single English NHS Trust serving predominantly inner city neighbourhoods. Comparison of unadjusted figures from our study with 2008–2009 Hospital Episode Statistics for England demonstrates possibly greater LOS in our service. For example, median length of stay in England for adult mental health services in this period was 17 days; for detained patients with schizophreniform psychosis it was 57 days [65]. As the effects of homelessness and residential mobility are presumably due to their effect on clinical decision-making, the extent to which these findings would generalise to other countries is uncertain; however, it is clear that homelessness and residential mobility themselves are common among psychiatric inpatients in most developed countries (see Introduction).

## Conclusions

Residential mobility and, to a lesser extent, homelessness appeared to have very significant effects on LOS within our sample. The effects are clearly detectable within the whole sample, but apply especially to the minority of individuals who are directly affected.

The starting point of any discussion of residential mobility should be to note that, considered in the round, voluntary residential mobility is the most important way in which people are able to adjust their housing to their needs [66], and the same is likely to apply to people to with and without a mental illness. However, evidence from studies of residential mobility among individuals with mental illness tends to suggest that forced, rather than voluntary, mobility may predominate, especially among individuals admitted to hospital: we have previously reported that wish to move and dissatisfaction with housing were unassociated with residential mobility in a population of individuals with severe mental illness [42]; furthermore, homelessness—which is unlikely to be voluntary—was strongly associated with residential mobility in the present sample [44], and the phenomenon of eviction at or around the time of hospital admission has previously been described [30,67]. Certainly, one future avenue for research would be to clarify the circumstances of inpatient residential moves and whether any proportion of them would have been better averted, investigations that would perhaps lead into better interventions to support tenancy sustainment.

Realistically, however, residential mobility is likely to remain a feature of a minority of psychiatric hospital admissions, and attention should also be focused on attempts to increase the supply of suitable housing units and to speed up the processes by which these are made available. Not only would this significantly reduce hospital bed-use (on the evidence of the present study) it would also mean that the individuals affected do not themselves have to suffer protracted and essentially unproductive admissions. Furthermore, the importance of residential mobility has significant implications for the design of psychiatric payment systems. Most obviously, systems that do not reimburse providers for the excess LOS of those awaiting new housing will impose very significant costs on providers, with the likely consequence that more patients will be discharged to unsuitable accommodation or discharged homeless. An alternative would be to attempt to reimburse costs imposed on providers— for example by paying a premium on top of the normal per case payment, or through making a per diem payment; but while this may avoid random risk to providers, it is a sub-optimal (allocatively inefficient) solution, as it would lead to greater resources being expended on healthcare, where this would yield little benefit, than on housing, where it would. The most efficient solutions are likely to be those that lead to a proper allocation of resources between healthcare and housing. In the UK, a system of cross-charging has been introduced whereby hospitals may impose fines on local authorities who do not provide appropriate community resources for elderly medical patients, and, although the effectiveness of this solution has been questioned [68], its extension to mental health services has been considered [18]. An alternative would be to pool housing and health resources, perhaps with health providers taking on the responsibility to provide housing for some of their users.

## Competing interests

The authors have no competing interests.

## Authors’ contributions

AT devised the study in association with PF and AD. AT constructed the data set, performed multiple imputation analysis (with assistance from MK), performed analysis (with support from MK) and wrote drafts of the manuscript. All authors reviewed and corrected the manuscript, and approved the final version.

## Pre-publication history

The pre-publication history for this paper can be accessed here:

http://www.biomedcentral.com/1471-244X/12/121/prepub
